# Methods for conducting international Delphi surveys to optimise global participation in core outcome set development: a case study in gastric cancer informed by a comprehensive literature review

**DOI:** 10.1186/s13063-021-05338-x

**Published:** 2021-06-21

**Authors:** Bilal Alkhaffaf, Jane M. Blazeby, Aleksandra Metryka, Anne-Marie Glenny, Ademola Adeyeye, Paulo Matos Costa, Ismael Diez del Val, Suzanne S. Gisbertz, Ali Guner, Simon Law, Hyuk-Joon Lee, Ziyu Li, Koji Nakada, Rafael Mauricio Restrepo Nuñez, Daniel Reim, John V. Reynolds, Peter Vorwald, Daniela Zanotti, William Allum, M. Asif Chaudry, Ewen Griffiths, Paula R. Williamson, Iain A. Bruce, on behalf of the GASTROS International Working Group, Shuangxi Li, Yu-long He, Zekuan Xu, Yingwei Xue, Han Liang, Guoxin Li, Enhao Zhao, Philipp Neumann, Linda O’Neill, Emer Guinan, Gian Luca Baiocchi, Giovanni de Manzoni, Eliza R. C. Hagens, Mark I. van Berge Henegouwen, Patrícia Lages, Susana Onofre, Gabriel Salcedo Cabañas, Maria Posada Gonzalez, Cristina Marin Campos, Bahar Candas, Bahadır Emre Baki, Muhammed Selim Bodur, Reyyan Yildirim, Arif Burak Cekic, Jean-Baptiste Beuscart, Sophie Horbach, Christopher Mecoli, Toby O. Smith

**Affiliations:** 1 Department of Oesophago-Gastric Surgery, Salford Royal Hospital, Salford Royal NHS Foundation Trust, Stott Lane, Manchester, M6 8HD UK; 2 Division of Cancer Sciences, School of Medical Sciences, Faculty of Biology, Medicine and Health, University of Manchester, Manchester, UK; 3 Centre for Surgical Research and Bristol and Weston NIHR Biomedical Research Centre, University of Bristol, Bristol, UK; 4 Paediatric ENT Department, Royal Manchester Children’s Hospital, Manchester University NHS Foundation Trust, Manchester, UK; 5 Division of Dentistry, School of Medical Sciences, Faculty of Biology, Medicine and Health, University of Manchester, Manchester, UK; 6 University of Ilorin Teaching Hospital, Ilorin, Nigeria; 7 Hospital Garcia de Orta, Faculdade de Medicina da Universidade de Lisboa, Lisbon, Portugal; 8 Basurto University Hospital, Bilbao, Spain; 9 Department of Surgery, Cancer Center, Amsterdam UMC, University of Amsterdam, Amsterdam, The Netherlands; 10 Department of General Surgery, Faculty of Medicine, Karadeniz Technical University, Trabzon, Turkey; 11 Department of Surgery, The University of Hong Kong, Hong Kong, China; 12 Department of Surgery and Cancer Research Institute, Seoul National University College of Medicine, Seoul, South Korea; 13 Peking University Cancer Hospital and Institute, Beijing, China; 14 Department of Laboratory Medicine, The Jikei University Daisan Hospital, Komae, Japan; 15 Hospital Universitario Rey Juan Carlos, Mostoles, Spain; 16 Department of Surgery, TUM School of Medicine, Munich, Germany; 17 Department of Surgery, Trinity Translational Medicine Institute and St James’s Hospital, Dublin, Ireland; 18 Hospital Universitario Fundación Jiménez Diaz, Madrid, Spain; 19 Regional Centre for Oesophago-gastric Surgery, Broomfield Hospital, Chelmsford, UK; 20 Department of Academic Surgery, Royal Marsden NHS Foundation Trust, London, UK; 21 Upper GI Unit, University Hospitals Birmingham NHS Foundation Trust, Queen Elizabeth Hospital Birmingham, Birmingham, UK; 22 MRC North West Hub for Trials Methodology Research, University of Liverpool and a member of Liverpool Health Partners, Liverpool, UK; 23 Division of Infection, Immunity and Respiratory Medicine, Faculty of Biology, Medicine and Health, University of Manchester, Manchester, UK

## Abstract

**Background:**

Core outcome sets (COS) should be relevant to key stakeholders and widely applicable and usable. Ideally, they are developed for international use to allow optimal data synthesis from trials. Electronic Delphi surveys are commonly used to facilitate global participation; however, this has limitations. It is common for these surveys to be conducted in a single language potentially excluding those not fluent in that tongue. The aim of this study is to summarise current approaches for optimising international participation in Delphi studies and make recommendations for future practice.

**Methods:**

A comprehensive literature review of current approaches to translating Delphi surveys for COS development was undertaken. A standardised methodology adapted from international guidance derived from 12 major sets of translation guidelines in the field of outcome reporting was developed. As a case study, this was applied to a COS project for surgical trials in gastric cancer to translate a Delphi survey into 7 target languages from regions active in gastric cancer research.

**Results:**

Three hundred thirty-two abstracts were screened and four studies addressing COS development in rheumatoid and osteoarthritis, vascular malformations and polypharmacy were eligible for inclusion. There was wide variation in methodological approaches to translation, including the number of forward translations, the inclusion of back translation, the employment of cognitive debriefing and how discrepancies and disagreements were handled. Important considerations were identified during the development of the gastric cancer survey including establishing translation groups, timelines, understanding financial implications, strategies to maximise recruitment and regulatory approvals. The methodological approach to translating the Delphi surveys was easily reproducible by local collaborators and resulted in an additional 637 participants to the 315 recruited to complete the source language survey. Ninety-nine per cent of patients and 97% of healthcare professionals from non-English-speaking regions used translated surveys.

**Conclusion:**

Consideration of the issues described will improve planning by other COS developers and can be used to widen international participation from both patients and healthcare professionals.

**Supplementary Information:**

The online version contains supplementary material available at 10.1186/s13063-021-05338-x.

## Introduction

A core outcome set (COS) is an agreed standardised set of outcomes that should be measured and reported, as a minimum, in all clinical trials in specific areas of health or healthcare [1]. COS should be relevant to key stakeholders and widely applicable such that researchers are encouraged and willing to incorporate them in trials. Approaches to improve the relevance of COS can take many forms, including involving stakeholders with lived experience of the condition or intervention in question. Many COS developers are using Delphi surveys during stages to prioritise potentially important outcomes [2]. A Delphi survey is a method of seeking consensus and asks participants to score items in terms of importance, usually using a Likert-type scale, across multiple survey rounds. In subsequent rounds, participants can reflect on their score and the ratings of others before being given the opportunity to change their scores if they wish. Using an online platform to undertake a Delphi survey enables overseas stakeholders to participate more readily in this process. Such broad participation can give COS greater validity across different geographical regions and consequently make them more likely to be used in future trials regardless of the location where trials are undertaken. Unless COS are widely used in trials within the same research field, the challenge of inconsistent outcome reporting will persist [3].

Most research groups developing ‘international’ Delphi surveys have restricted themselves to their native language (usually, but not exclusively, English). This approach is less resource intensive than translating the survey into multiple languages and overcomes issues with ambiguity or changes in meaning—a recognised challenge with translation [4]. However, these methodological challenges are not insurmountable, and some COS developers are translating Delphi surveys to minimise the risk of excluding important opinion from those not fluent in the study’s primary language.

The GASTROS study (*GA*stric *C*ancer *S*urgery *TR*ials *R*eported *O*utcome *S*tandardisation) aims to develop an international COS for surgical trials in gastric cancer [5]. The scope and design of the GASTROS study have been previously detailed [5]. In summary, following a systematic review of randomised control trials [3] and a series of in-depth patient interviews [6], a long-list of potentially important outcomes was rationalised into a list of 56 outcomes. Following a consultative exercise with key stakeholders, these 56 outcomes were presented to patients and healthcare professionals in a two-round, multi-language Delphi survey. Currently, there is no standardised method of translating Delphi surveys for use in the development of international COS. This paper aimed to address this need by using GASTROS as a case study to implement a methodological approach to translation developed from international consensus guidelines in the field of outcome reporting [4].

### Objectives

The objectives of this paper include:

1. To describe the current methodological approaches used by COS developers in the translation of Delphi surveys;
2. To outline a pragmatic, robust and replicable approach to translating Delphi surveys for use in COS development; and
3. To outline important logistical considerations in preparation for an international Delphi survey.

## Methods

### Assessing current approaches to translating Delphi surveys (methodology)

To gain an understanding of current translation approaches for multi-language Delphi surveys, a comprehensive literature review of the COMET database was undertaken [7]. The COMET database is a comprehensive registry which (as of 03/09/2019) contained 337 published and 280 ongoing COS respectively dating back from 1981. The database is kept up to date through annual systematic reviews of scientific databases (using MEDLINE via OVID and SCOPUS); automated alerts from MEDLINE via OVID, SCOPUS and Google Scholar; and direct submissions from COS developers [2].

### Search strategy and inclusion criteria

The COMET database enables users to search for terms within the ‘title’, ‘abstract’ or ‘author names’ categories. Searches can be restricted according to health area, target population, methods, stakeholder involvement, study type and publication year. A broad search for the terms ‘international’, ‘language’ or ‘translat$’ in the title and abstract was undertaken with no other restrictions.

Studies included in our review were those that used a multi-language Delphi survey in the development of their respective COS. Only publications from completed studies were included—COS methodology is a relatively new research field and so planned approaches may not accurately reflect the final methodology used. The COMET database may contain several different references to COS development for the same project. Any related publications were consolidated and handled as a single COS study.

Corresponding authors were contacted and asked to participate in a questionnaire examining various aspects of their respective methodological approaches (Additional file 1). The questionnaire focussed on how items presented in the Delphi surveys were translated and how discrepancies and conflict were resolved. Responses were received from the corresponding authors of all studies identified and combined with data from the respective publications.

### Approach to translating the GASTROS Delphi survey

One of the principal aims of translating the Delphi survey in the development of COS is to include the opinions of stakeholders who are not fluent in the source language. With respect to the GASTROS study, this was especially important given that the highest incidence of gastric cancer exists outside of English-speaking countries, in the Far East, Central and South America and Southern Europe.

In developing our approach to translate the survey, the study management group was keen to ensure that it was both methodologically sound yet pragmatic such that it could be easily reproduced by multiple international collaborators within a relatively short period of time.

In 1999, the ISPOR-TCA group (The Professional Society for Health Economics and Outcomes Research – Translation and Cultural Adaptation group) was formed to discuss and develop guidelines for translating patient-reported outcome measures. The group highlighted inconsistencies with previous methodologies and nomenclature in this field and sought to address these by developing guidance setting out ‘principles of good practice’ [4]. These principles were derived from 12 major sets of translation guidelines from the following groups:

- American Association of Orthopaedic Surgeons (AAOS)
- Association of Test Publishers
- EORTC group
- Euro QoL group
- Evidence: Clinical and Pharmaceutical Research
- FACIT group
- Health Outcomes group (HOG)
- Health Utilities Inc. (HUInc)
- International Quality of Life Assessment (IQOLA) group
- Kidney Disease Quality of Life (KDQOL)
- Medical Outcomes Trust (MOT)
- World Health Organization

Other consensus guidelines have been developed for translating surveys. The Survey Research Centre (SRC) guidelines provide broader consideration of the translation process and describe practical support from expert contributors’ experience of different survey types [8]. There is much cross-over between the two guidelines. Given the focus of our work was primarily outcome-related translation, the principles as set out by the ISPOR-TCA group formed the basis of our methodology, with references made to the SRC guidance and some pragmatic amendments which are explained in further detail below.

### Eligibility criteria for target languages

The target languages were chosen to enable increased recruitment from regions with a significant incidence of gastric cancer and experience of research activity within this field. The source survey was developed in English and translated into seven target languages (Simplified Chinese, Dutch, German, Italian, European Portuguese, European Spanish and Turkish). By facilitating participation from these regions, we aimed to improve the validity of our COS such that it would be more likely to be used by researchers in future trials.

## Results

### Comprehensive literature review of previous translation approaches

**Fig. 1:**
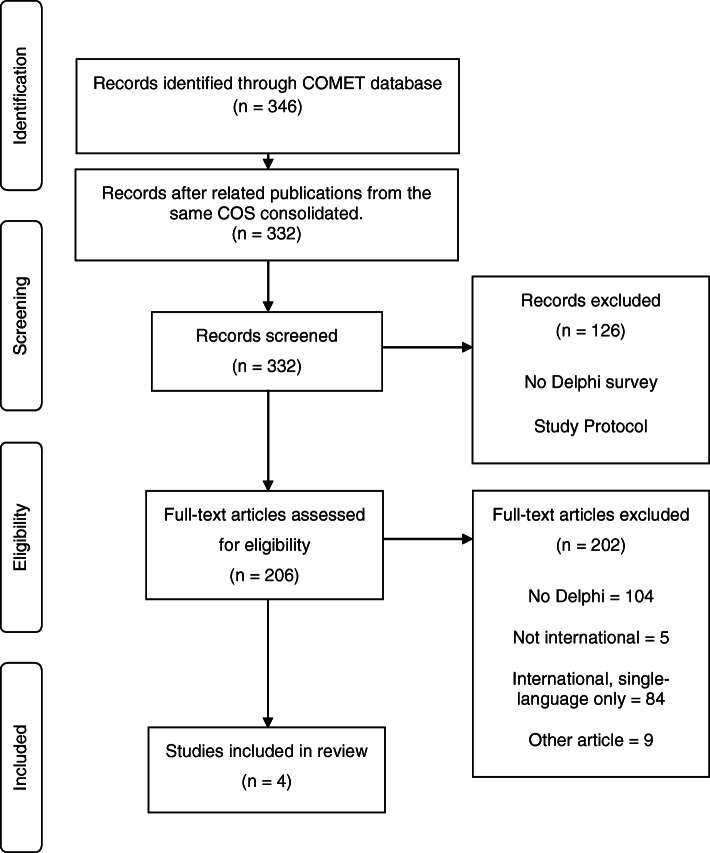
Flow diagram demonstrating which studies were included in the systematic review

Three hundred forty-six records were identified from the COMET database from which four studies (summarised in Table 1) were deemed eligible for inclusion in the comprehensive literature review. The process through which these were identified is summarised in Fig. 1.

**Table 1:** Studies using multi-language Delphi surveys in the development of international COS

Condition/group	Original language	Target language(s)	Total participants in surveys	Total participants using translated survey(s) (%)
Hip and knee osteoarthritis OMERACT-OARSI [9]	English	Italian and Spanish	426	2 (0.5%)
Medication review in multi-morbid older patients with polypharmacy OPERAM [10]	French	Dutch, German, English	150	118 (79%)
Idiopathic inflammatory myopathy OMERACT [11]	English	Swedish, Dutch and Korean	500	120 (24%)
Vascular malformations OVAMA Group [12]	English	Dutch	301	72 (24%)
*Gastric cancer* *GASTROS study*	*English*	*Chinese, Dutch, German, Italian, Portuguese, Spanish, Turkish*	*952*	*637 (66%)*

#### General approaches to translation currently in use

**Fig. 2:**
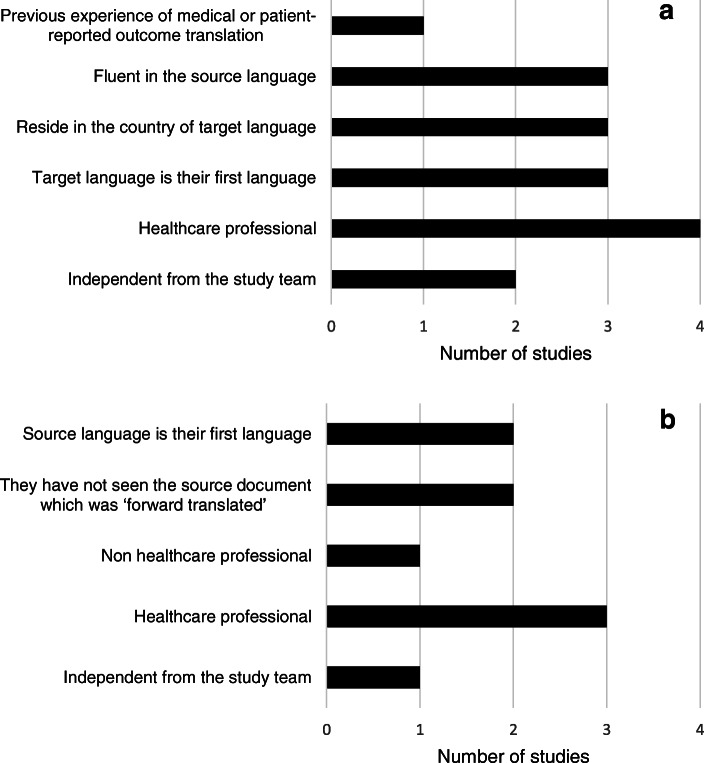
**a** Characteristics of translators undertaking forward translation(s). **b** Characteristics of translators undertaking backward translation(s)

All 4 COS groups summarised their approach to translating surveys with one providing a reference to their methodology and another referring to the methodology described by the OMERACT group at a COS development meeting [8, 13]. In addition to forward translations, three groups undertook a backward translation of the survey from the target to the source language. The number of forward and backward translations differed in each study. Two studies undertook a single forward translation whilst the others undertook two and three. One group used no backward translations and one study undertook a single backward translation whilst the other two undertook two backward translations. The characteristics of those involved in the translation processes also differed amongst the groups (Fig. 2); no paid translation services were employed, and all translations were undertaken by healthcare professionals or lay translators.

#### Discrepancies and harmonisation

All four groups described an approach to managing discrepancies in translations. Two groups reported that discrepancies were discussed within the ‘research group’ until consensus was reached, whilst the remaining two referred to individuals outside of the ‘research group’ who were fluent in the target language to resolve any language issues.

#### Cognitive debriefing

Three groups described undertaking an exercise to test alternative wording and check understandability, interpretation and cultural relevance of the proposed Delphi survey in the target language. Using interviews, they studied patients/relatives and health professionals’ interpretation of the translations to examine face validity (the degree to which the survey appears effective in terms of its stated aims). Two of these involved patients and/or their relatives whilst the third was based on the opinion of healthcare professionals alone.

### Results from the GASTROS study

The GASTROS study was able to recruit 952 eligible participants (445 surgeons, 268 patients, 239 nurses) in the first round of the Delphi survey, with 315 participants using the English-language version and 637 using one of the seven other language versions (Table 2). Sixty-two per cent (166/268) of patients used translated surveys compared to 69% (471/684) of healthcare professionals (62% of surgeons and 82% of nurses).

**Table 2:** Uptake of translated Delphi surveys in non-English-speaking regions

Regional language	Patients (***n*** = 268)		Surgeons (***n*** = 445)		Nurses (***n*** = 239)	
	Translated version	English version (%)	Translated version (%)	English version (%)	Translated version (%)	English version (%)
Chinese	60 (97%)	2 (3%)	109 (97%)	3 (3%)	109 (100%)	0 (0%)
Dutch	5 (100%)	0 (0%)	22 (100%)	0 (0%)	10 (100%)	0 (0%)
German	4 (100%)	0 (0%)	10 (100%)	0 (0%)	–	–
Italian	57 (100%)	0 (0%)	57 (95%)	3 (5%)	12 (100%)	0 (0%)
Portuguese	1 (100%)	0 (0%)	28 (88%)	4 (12%)	8 (100%)	0 (0%)
Spanish	–	–	33 (94%)	2 (6%)	0 (100%)	0 (0%)
Turkish	39 (100%)	0 (0%)	17 (94%)	1 (6%)	56 (100%)	0 (0%)
Other language^a^	No translation undertaken	0	No translation undertaken	97	No translation undertaken	13
Total	166 (99%)	2 (1%)	276 (96%)	13 (4%)	195 (100%)	0 (0%)

Percentages reported refer to the proportion of participants from the respective region within each stakeholder group

^a^‘Other language’ refers to regions where English was not the first language, but where the survey was not translated

#### Development of translation approach

Below, we describe ten steps involved in translating the Delphi survey used in the development of a COS for surgical trials in gastric cancer. The full rationale for each step, and the risks of omitting them, is described in detail in the ISPOR-TCA guidance; we have stated the rationale for the steps below (particularly in relation to pragmatic deviations) where we believed it was necessary to do so.

Additional file 2 details the instructions which were provided to each international collaborator responsible for leading the translation process in their respective country. These outlined which files required translation, how the translation should be undertaken and by whom.

##### Step 1: Preparation

**Fig. 3:**
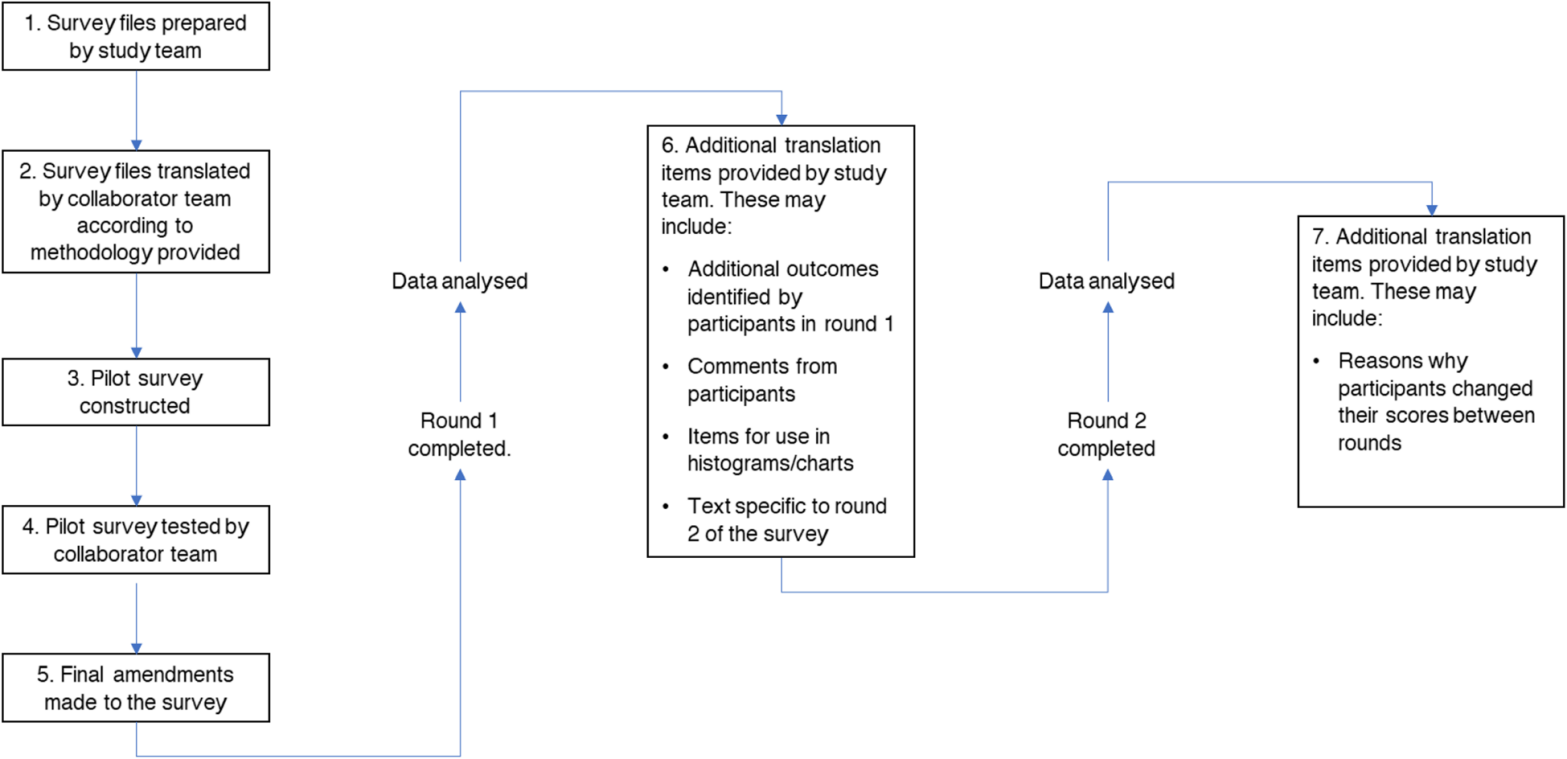
Step-by-step translation process for multi-language Delphi surveys

- Cognitive debriefing:
  i. Cognitive debriefing describes a process which aims to identify issues with comprehensibility of key concepts and understanding amongst potential participants. As previously stated, we presented survey participants with 56 outcomes which had been rationalised following a process that had identified a long-list of potentially important outcomes from a systematic review and in-depth patient interviews. The rationalisation process from the long-list to the 56 survey items involved key stakeholders (members of the GASTROS study group, surgeons, oncology nurses and patients) who also ensured that the outcomes were accompanied with plain English-language explanations that could be understood by all participants including patients. A further consultative exercise with an English-speaking patient-group was held to ensure that the meaning of each outcome, in addition to other survey-related files, was clearly understood. Undertaking this work prior to translation was essential as it minimised the possibility of ambiguous meanings which could result in a mistranslation.
- Preparing documents for translation
  i. Four documents were needed to run the Delphi survey: a participant information sheet and three further files which were required to set up the web-based survey. We used DelphiManager 3.0 platform, developed and maintained by the COMET Initiative (http://www.comet-initiative.org/), to undertake the Delphi survey (see the ‘Important considerations’ section). A comprehensive overview of the platform’s functionality and capability can be found at https://www.comet-initiative.org/delphimanager/. The three files included:
    1. File 1 (Additional file 3): an Excel file containing details of each outcome, accompanying meaning and the ‘outcome area’ under which the outcome was categorised [14].
    2. File 2: user-defined text: a file containing text specific to our surveys (in this case the GASTROS Delphi survey).
    3. File 3: static text: a file containing text common to all Delphi surveys which was used in the setting up process by the DelphiManager team.
  ii. Preparation for round 2 of the Delphi survey: additional translations were required to support the second round of the survey. These included:
    1. Outcomes identified by participants in round 1 as being important to consider that were not identified from the systematic review or patient interviews.
    2. Legends and terms required to produce charts which were presented to survey participants in round 2.
    3. Comments and feedback from study participants.
  iii. Following round 2 of the survey:
    1. Participants who changed their scores between rounds were given the opportunity to provide their reasons for doing so.
    2. Participants were also given the opportunity to provide further comments after completing the survey.
- Understanding which methodological approaches to employ
  Due to the resources required for different methodologies, we opted for two approaches to translation. Our rationale for applying each approach is described below:
    1. ‘Two forward, one back translation’; the terms ‘forward’ and ‘backward’ refer to the direction of translation between the source and target languages, with forward referring to a translation from the source language and backward referring to a translation from the target language back to the source language. This approach was the most comprehensive and labour intensive as it required a further nine steps (below) before a final file version was agreed. Following discussion amongst the study management team, it was deemed content which could alter the meaning of the outcomes being presented and ultimately influence how the overall aims of the survey was received and understood by participants (file 1, file 2 and additional outcomes identified by participants in round 1) underwent this approach. The steps involved in this process are described in greater detail in points 2 to 10.
    2. ‘One forward, dual independent proofreading’; file 3 consisted primarily of short instructional phrases (e.g. ‘click here’, ‘register’ and ‘next page’) which were necessary for the functionality of the survey. As these terms would not materially influence the comprehension of the survey’s purpose or outcomes presented within it, a simplified, less resource-intensive approach was adopted. This file underwent a single forward translation followed by two independent proof-readings by translators who compared the translated and source files for accuracy and quality. Any corrections or amendments were undertaken through discussion between the translator and proof-readers. This approach was also adopted for the translation of participant comments, feedback and reasons for changing scores between round 1 and round 2.
- Setting up translation teams
  To support the translation work, an international working group (IWG) was established (see the ‘Important considerations’ section). Each collaborator within the IWG was responsible for overseeing a team which would undertake the translation and ensuring that the key concepts of the study were appropriately communicated. The translation process was supported by the GASTROS study Chief Investigator (BA) if any clarifications were required. The characteristics of individuals involved in this process are described in greater detail in Additional file 2. In summary, each team was made up of an IWG lead, two forward translators and a single backward translator.
- Developing instructions for translations
  Setting out the methodology a priori in a clear and structured document ensured that collaborators and their teams understood what would be required of them at each stage of the translation. These instructions included ongoing responsibilities prior to and following future rounds of the Delphi survey. This was essential given that one of our primary aims was to ensure that our approach was easily replicable. Figure 3 is a flow diagram which details these stages and the order in which they were to be undertaken. Feedback from the IWG was positive in response to these instructions with collaborators reporting that the document enabled them to undertake the translation process efficiently.
- Quality assurance
  IWG collaborators were asked to provide documented evidence for each step of the translation process. These could then be reviewed by the study management team as required.

##### Step 2: Forward translation

Two independent forward translations by individuals who were native speakers of the target language were undertaken. Culture is a primary determinant of language and therefore native speakers have advantages with language abilities compared to second-language speakers. Having two independent forward translations enables detection of errors and divergent interpretations that could otherwise lead to bias.

##### Step 3: Reconciliation

There are several approaches which can be used to reconcile the forward translations. We opted to use the ‘in-country’ IWG collaborator who was also involved in cognitive debriefing and piloting of the survey as this was pragmatic and would not require the identification of further individuals to undertake this step. No issues arose from the reconciliation process; however, had further clarifications been required, they would have been directed to the Chief Investigator (CI).

##### Step 4: Back translation

The issue of whether ‘back translation’ is required is one on which there is disagreement; the ISPOR-TCA guidance states that ‘back translation’ is necessary, whilst the Survey Research Centre guidance suggests that it is not. COS developers *may* therefore be justified in omitting steps 4 and 5 of our approach. This should however be done after careful consideration as the importance of back translation may depend on the type of outcomes that are being translated. It is possible that certain outcomes are conceptually alien between cultures or geographical regions and undergoing an added step to reduce the risk of mistranslation is warranted. In the field of patient-reported outcome measurement (PROM), it is common for questionnaires to undergo translations (for use in international trials). The methods required for PROM translation are rigorous and include back translation [15]. Whilst it may be argued that less rigorous methods could be used in Delphi surveys for COS, to ensure optimal face validity of items, the same standards are recommended.

We opted to undertake a single back translation to provide quality control of the forward translations. Whilst the ISPOR-TCA guidance suggests that this should be undertaken by individuals who are native speakers of the source language (i.e. English), we found it challenging to identify seven native English speakers who were also fluent in the required target languages and had an understanding of outcome reporting without referring to a professional service (paid professionals with expertise in translation). We opted to ensure that back-translators were fluent in English and independent from the forward translators.

##### Step 5: Back translation review

This step is important as it ensures that the cross-cultural adaptation needs of the translation are met. Cross-cultural adaptation ensures that the imprinted knowledge, attitudes, values, perceptions and behaviours of different regions are accounted for in the understanding of the terms being translated. Without it, there is a risk of that a mistranslation or omission would remain in the translation. This was undertaken by the CI in combination with the IWG collaborator by comparing the back translation to the source document. No significant discrepancies between the source and back-translated files were identified across any of the translations.

##### Step 6: Harmonisation across different languages

There is no agreed method to how harmonisation across different translations should be enforced; many approaches omit this step. However, our group opted to ensure harmonisation between each language at each step of the process. This was undertaken by the CI. We did not encounter significant differences between translations. An example of a minor change that was made across surveys was the term ‘last round scores’ which in the context of the survey meant ‘*previous* round scores’. Some teams translated this as ‘the final round scores’ which had to be altered to ensure all versions contained the same meaning.

##### Step 7: Cognitive debriefing of the translation

Following harmonisation across translations, all survey versions were built using the DelphiManager platform (see the ‘Important considerations’ section). A further cognitive debriefing exercise was undertaken by asking IWG collaborators and their translation teams to complete a pilot version of the survey to identify grammatical or stylistic errors and check understandability, interpretation and cultural relevance of instructions and outcomes within the survey.

##### Step 8: Review of cognitive debriefing results and finalisation

There were no issues highlighted with comprehensibility or understanding. Spelling mistakes and minor grammatical errors (e.g. pronouns ‘you’ formal and informal) were altered.

##### Step 9: Proofreading

IWG collaborators were once again asked to examine the survey and ensure that any issues highlighted in the previous steps had been addressed. No further changes were identified in any of the language versions by this stage.

##### Step 10: Final report and ‘start of survey’

The ISPOR-TCA group guidance recommends that a report should be produced detailing the methodological approach for translation and rationale for each step. The final report for translations undertaken for the GASTROS study is represented by this paper. The complete survey presented in round 1 of the Delphi survey is presented in Additional file 4.

#### Important considerations

Whilst applying the described approach to translating the GASTROS Delphi survey, several key issues were identified that are summarised in Table 3 and described in greater detail below. These should be considered alongside the translation work to maximise recruitment. We describe the rationale for each consideration and the potential risks of not applying these steps (where applicable).

**Table 3:** Nine key considerations for COS developers undertaking multi-language Delphi surveys

1 International working group	To ensure that study and its aims are promoted in regions from where the study team wish to target recruitment.
2 Patient and public involvement	To ensure that the patient perspective is represented.
3 Who should undertake the translation work?	Deciding whether to employ professionally paid services or identify clinically trained individuals to undertake the translations.
4 Milestone and timeline planning	Providing a pre-agreed timetable for translation work and checks ahead of recruitment to the Delphi survey.
5 Recruitment and retention targets	Planning how long to keep Delphi survey rounds open to ensure an appropriate number of participants have been recruited.
6 Paper and Internet-based survey versions	Giving stakeholders without easy access to the Internet an opportunity to participate in the study.
7 Measures to maximise recruitment	Dissemination strategy
	Local recruitment
	Support from stakeholder group and research networks
	Collaborations
	Personalised e-mails
	Social media and multimedia
8 Ethical approval	Identifying what type of approvals are required as these vary between regions.
9 Financial planning	Ensuring that a robust plan for resource allocation is made in advance.

##### International working group

The GASTROS study is a collaborative international initiative which sought to attract global representation within the study group. Motivated, research-active collaborators from countries with a significant incidence of gastric cancer were approached to form an IWG. Individuals signed a ‘terms of reference’ document which outlined the benefits of their involvement in addition to the following responsibilities:

- To form a local team and oversee the translation of the Delphi survey (where applicable)
- To drive recruitment locally, regionally, nationally and internationally through organisations and personal networks
- To garner and develop links specifically with patient groups who would be able to participate in advertising the Delphi survey
- To identify the need and apply for relevant local ethical and regulatory approvals

The IWG was made up of collaborators from the following countries:

- Brazil
- Mainland China and Hong Kong
- Germany
- Ireland
- Italy
- Japan
- The Netherlands
- Nigeria
- Portugal
- South Korea
- Spain
- Turkey
- UK

Ensuring the IWG was set up early maximised our ability to develop translations in a timely manner and recruit evenly across all stakeholder groups from a broad range of countries.

##### Patient and public involvement

A Study Advisory Group (SAG) separate to the IWG formed part of the management structure of the wider GASTROS study. The SAG was made up of key stakeholder representatives including patients. The group provided advice on the methodology of the study and general delivery of the study against its stated objectives and ensured that the viewpoints of all stakeholder groups were considered. In addition, patient groups (see the ‘Acknowledgements’ section) were vitally important in reviewing and piloting the translated surveys prior to recruitment to the Delphi. These groups were also instrumental in recruiting patients (see below).

##### Who should undertake the translation work?

The GASTROS study management group opted to set up local translation teams made up of healthcare professionals who met the rigorous criteria as set out by the ISPOR-TCA group. An alternative approach would have been to employ a professional translation service to undertake this work. One of the benefits of professional services is the ability to complete the translations in a relatively short period of time, in addition to developing an unlimited number of translations which may have resulted in wider participation in the Delphi survey. The main disadvantage to this approach is cost. Quotes from three different professional translation services (all familiar with the ISPOR-TCA guidance) were requested to support rounds 1 and 2 of the survey. In April 2018, the estimated costs were in the region of 3200GBP–4000GBP per language. All translations for rounds 1 and 2 of the survey would be finalised within 5 and 2 weeks respectively. Due to the financial limitations of undertaking the survey in 7 languages, we did not pursue this option.

##### Milestone and timeline planning

A summary of the resulting timelines involved in producing all versions of the survey using our approach to translation is provided in Table 4. Setting aside enough time for the translation process is of paramount importance, particularly if COS developers are seeking to translate their surveys into more than one language. Some of the translation steps required all language versions to have reached the same stage prior to moving onto the next stage. For example, all initial translations had to have been completed before harmonisation across surveys could be achieved. Without this, we were unable to ask collaborators and their teams to pilot their respective surveys. Furthermore, we chose to open recruitment to all language versions simultaneously and so all translations needed to have been fully completed before participants could complete their surveys. This was also the case for the second survey round. The impact of ethical approval applications on timelines is discussed below in greater detail. The time to return the initial translation documents and obtain ethical approvals resulted in the greatest variations with respect to the overall timelines. We found that setting regular milestones and realistic timelines helped achieve the required translation objectives. Regular communication between the CI and collaborators underpinned this process.

**Table 4:** Timeline-related considerations in undertaking multi-language Delphi survey in the GASTROS study

Language version^**a**^	Document preparation	Time to return completed translations for r1	Harmonisation across language versions	Time to set up online surveys	Time to pilot survey and complete amendments	Time to obtain ethical approval^**b**^	Time r1 open	Time to analyse results from r1 and produce additional translation files	Time to return translation documents for r2	Time r2 open
Translation 1	2 weeks	6 weeks	2 weeks	1 week	1 week	25 weeks	13 weeks	3 weeks	2 weeks	12 weeks
Translation 2		10 weeks			1 week	29 weeks		3 weeks	1 week	
Translation 3		3 weeks			1 week	Not required		3 weeks	1 week	
Translation 4		10 weeks			1 week	Not required		3 weeks	1 week	
Translation 5		18 weeks			1 week	^c^Not received		3 weeks	1 week	
Translation 6		12 weeks			3 weeks	40 weeks		3 weeks	1 week	
Translation 7		2 weeks			1 week	2 weeks		3 weeks	1 week	

^a^The language versions are anonymised

^b^This represented the time the study management group requested collaborators to begin ethical approval applications until IRB approval was received and not necessarily the time between actual submission of the application and receiving approvals

^c^Ethical approval was not received before the end of round 1 of the Delphi survey. No patients were recruited from this team’s country

Our aspiration was to translate the Delphi survey into Japanese and Korean to enable wider patient participation from these countries. Due to challenges in identifying collaborators at an early stage, assembling a translation team and meeting timelines, this could not be pursued. However, potential participants were invited to complete the English-language version of the survey.

##### Recruitment and retention targets

**Fig. 4:**
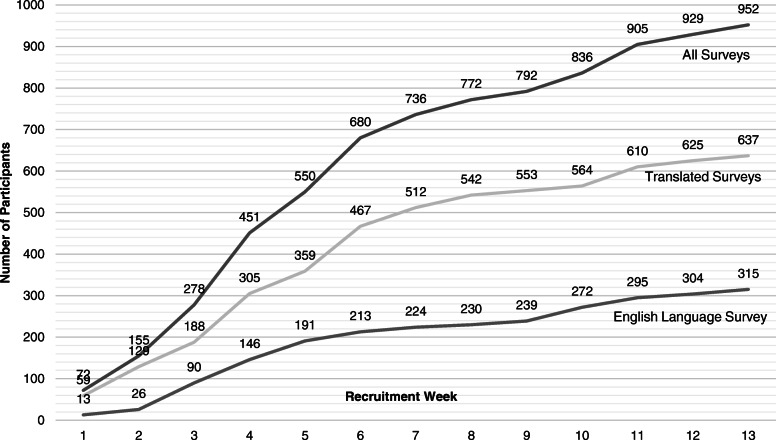
Cumulative weekly recruitment figures for round 1 of the GASTROS Delphi survey

COS developers should consider minimum recruitment targets. Whilst there is no sample size requirement for Delphi surveys, the GASTROS protocol initially set a conservative target of 100 participants in total to be recruited over a period of 6 to 8 weeks in round 1. However, as interest in the study and international collaboration grew, it was clear to see that this target would easily be surpassed. As described below, once the survey opened and momentum began to gather, we witnessed a ‘snowballing’ effect amongst all three stakeholder groups. We therefore extended recruitment to 13 weeks by which time new participation had plateaued (Fig. 4).

For round 2, an initial retention target of 80% was set following discussions with members of our study management group who have extensive knowledge and experience of COS development. Automated reminder e-mails were sent out on a weekly basis to participants and support from professional bodies (in countries where round 2 responses were slow) was sought to encourage completion of the survey. Personalised e-mails from the CI to professionals were also sent. Using this strategy, we were able to retain 70% of participants from round 1 by week 13, by which time no further responses were being received.

##### Paper and Internet-based Delphi survey versions

The GASTROS study used both Internet-based and paper versions of the Delphi survey. The internet-based versions enabled us to reach participants in nearly 60 countries, the vast majority of which did not have formal IWG collaborators. The paper versions (printed versions of the Internet-based survey which were uploaded electronically by local collaboration teams) also enabled us to recruit participants (particularly patients) who either did not readily have access to the Internet or were not ‘electronically-literate’.

Several platforms exist to enable COS developers to run Delphi surveys. These include platforms specifically designed for Delphi surveys and other generic survey platforms which researchers can use. When considering multi-language surveys, it is essential to ensure that the servers on which the surveys are hosted meet the necessary data protection regulations and are accessible particularly from countries where restrictions to certain domains exist. Furthermore, COS developers must ensure that the platforms used are able to run surveys using different language scripts and writing systems.

Our group used DelphiManager as it fulfils the required data protection criteria (as set out by our UK ethical approval) and can work with all language systems including English, Chinese, Japanese and Korean. Furthermore, the online survey domains are accessible from countries which commonly restrict access to other foreign domains. DelphiManager has additional features which simplified recruitment and completion of the surveys such as being able to send automated reminders to individuals who had yet to complete all their answers.

##### Measures to maximise survey recruitment

One of the strengths of our Delphi survey was that it was able to recruit approximately 1000 eligible patients and healthcare professionals from nearly 60 countries in round 1. From the study’s inception, the study team recognised the importance of developing a clear networking and dissemination strategy [5]. We hypothesised that this was necessary to achieve broad stakeholder participation both nationally and internationally. Several strategies were employed to maximise recruitment:

**Dissemination of results from previous study stages**

The study protocol and findings from previous study stages [3, 5, 6] were presented at targeted national and international meetings which were well-attended by potential healthcare worker participants. This was integral to generating interest and support for our study and ensured that participants understood the premise for GASTROS long before the Delphi survey opened for recruitment. All presentations contained directions to the study website and social media accounts (see below).

**Local recruitment of patients through outpatient clinics**

Ethical approval enabled the study team to recruit patients directly from outpatient clinics. Our experience from the UK is that many patients regularly attend patient support groups and are in contact with other eligible patients. As a result, a snowballing effect resulted in patients being recruited by patients already within the study.

**Support from stakeholder groups/associations and national research networks**

Support from national and international professional associations and organisations was sought in the early stages of the study. Study group members presented the study objectives at closed executive-level meetings to gain support and adoption from influential bodies including professional associations, patient groups and charities. Many of these organisations have large memberships (and corresponding electronic mailing lists) through which the study was advertised. Most of the groups through whom we sent out invitations followed up an initial e-mail with a further reminder approximately 4 weeks later resulting in further recruitment spikes. Furthermore, the GASTROS study was adopted onto the National Institute for Health Research (NIHR) Portfolio (CPMS study ID 38318). This enabled us to advertise the study to healthcare professionals and patient support groups within the UK through the national Clinical Research Networks. Our experience suggests that recognition by respected associations and groups results in a ‘snow-balling’ effect with subsequent support from others becoming easier to harness.

**Collaborations**

Standardising the reporting of outcomes can be achieved through several approaches. The GASTROS study aims to identify important outcomes across the entire spectrum of outcome types. Others have concentrated on the reporting of outcomes within a defined area. For example, the Gastrectomy Complications Consensus Group (GCCG; www.gastrodata.org) has sought to standardise the reporting of all major post-gastrectomy complications [16]. Whilst the goals of both studies are different, both teams have been able to work closely to minimise duplication of work. In addition, the GCCG was able to promote recruitment to the GASTROS Delphi survey through its membership and respective networks. Such collaborations will also be vital for the future development of outcomes research within the field of gastric cancer surgery.

**Personalised e-mails**

- Most of the study management group, study advisory group and international working group members have extensive research experience within the field of gastric cancer surgery. Each member was asked to promote the study through their personal research and clinical networks. Bulk e-mails through professional bodies may be ignored by potential participants or diverted into ‘spam’ e-mail folders, hence why this approach was employed.
- Corresponding e-mail addresses for authors from previous trials and protocols included in our systematic review [3] were identified and personal invitations sent. This captured research-active healthcare professionals from non-English-speaking regions where no formal national gastric cancer associations exist (e.g. Eastern Europe).

**Study website, social media and multimedia**

- The study website (http://www.gastrosstudy.org) provides detailed information about the GASTROS study aims as well as all its outputs. Prior to the commencement of the Delphi survey, potential participants who had heard about the study were able to register their interest to participate in the Delphi survey. In the preceding 18 months before the survey opened, 150 healthcare professionals and patients had registered.
- In addition to the study’s Twitter account (https://twitter.com/GASTROSStudy), members of the research team posted updates on their personal Twitter and LinkedIn accounts. Regular study updates provided potential participants with an opportunity to better understand the study aims and keep up to date with its progress. Examination of analytics revealed that Twitter and LinkedIn posts in the run-up to and during round 1 of the survey regularly received over 4000 and 3000 views, respectively.
- A series of short videos were produced for the study. These provided potential participants with an alternative way to engage with the study. At the time of writing, these videos had been viewed over 600 times. In addition to an introductory video on the study, a detailed step-by-step guide to completing the online Delphi survey was developed. This created additional content for social media platforms and the GASTROS website which in turn enabled the study to maintain a regular online presence. COS developers may wish to produce different language versions or translate video captions relatively easily to expand their reach. Additional COS-related material is already available from the COMET initiative YouTube site [17] with versions available in Dutch, Portuguese and Chinese. Work is underway to develop other language versions as well.

Whilst advertising the study through these avenues increased the number of recruits, care must also be taken that potential participants are not ‘bombarded’ with requests to participate in the survey. A small number of healthcare professionals highlighted that this was an issue. This coupled with the well-recognised challenges of ‘survey-fatigue’ may in fact be counter-productive and result in apathy amongst potential participants.

##### Ethical approval

The requirement for regulatory or ethical approval varied across different regions. In the UK, the approach to ethical approval has not been consistent; our group was asked to submit a full application for ethical approval committee consideration, whilst other groups have been able to gain approval through proportionate review [18]. Each IWG collaborator was responsible for understanding local requirements and applying for approvals if they were required. They were asked to enquire about these at the start of their agreement to participate in the study and applications were made in parallel to the translation work. Two of our international collaborating centres did not require ethical approval as local collaborators did not recruit patients directly from their clinical practice but instead advertised the study through local patient groups and recruited healthcare professionals by advertising through national Societies and networks. The time taken to complete this process varied significantly (Table 4) and was largely dependent on the frequency of and access to ethics committee meetings, requirements to amend submitted materials and delays in final decisions reaching the collaborators. COS developers should investigate the need for ethical approval as early as possible to avoid unnecessary delays.

##### Financial planning

Several aspects of undertaking multi-language Delphi surveys may incur significant costs depending on which approaches are adopted. COS developers should take these into account when planning their studies. These include:

1. Cost of professional translations; this represents the largest financial burden and has been discussed above.
2. Ethical and regulatory approvals; some of our non-UK ethical approval applications required payments of up to 250 Euros.
3. Use of electronic mailing lists; some stakeholder groups may charge administration fees to send out invitations to their membership.
4. Cost of Delphi survey platform; whilst open-access platforms exist, our group opted to pay to use a dedicated Delphi survey platform designed for the development of COS.
5. Statistical and qualitative methods support may be required when analysing scores in rounds 1 and 2, depending on the nature of feedback to be given.

## Discussion

This study is the first to address the topic of translation and cross-cultural adaptation in the context of developing Delphi surveys for COS. We have presented a detailed and easily reproducible approach adapted from international consensus guidelines which was illustrated within the context of developing of a COS for gastric cancer surgery. The approach was accurately replicated by seven different translation teams within an acceptable timeframe.

Undertaking ‘international’ Delphi surveys has become easier as web-based platforms enable wider participation across different geographical regions. Whether or not this is warranted depends on the scope and target audience of the COS in question. As very few pathologies and interventions are limited to one geographical region, most clinical trials are undertaken globally. For these trials to include outcomes relevant to all stakeholders, trialists need to be confident that the COS relevant to their fields are robust. In our example, stakeholders from Asia, South America and Europe were essential to the development of the COS as gastric cancer is most prevalent in these countries and most trials are undertaken in these regions. The resulting number of participants from non-English-speaking nations was significantly higher than those recruited from English-speaking countries. It was therefore important that processes used for ensuring accurate translations were valid and transparently reported.

Whilst translation of our Delphi survey aimed to widen participation and broaden the views taken into consideration, the value of doing so is one which warrants further discussion. English is the most commonly spoken language across the world [19] and is used by most scientific and healthcare publications. It is therefore unsurprising that most ‘international’ COS projects employing Delphi surveys identified from the COMET database used an English-only version. The English version of our Delphi survey was offered to all participants; however, most preferred to complete the Delphi in their native language. This was the case for both patients and healthcare professionals. Patients were primarily recruited from regions in which surveys were translated to the local language. Whilst for many the choice of using a non-English survey version would have been because they did not speak English, a significant proportion of bi-lingual healthcare professionals known to the study team preferred to use a non-English version. One may argue that this enabled participants to engage more confidently in the process, that their understanding of what was being asked of them was clearer and that the quality of their responses may consequently have been better.

Such a high uptake in non-English surveys was not experienced by three of the four groups who completed our questionnaire on methodology. This was also reflected in certain subsets of stakeholder groups which had access to translated surveys in the GASTROS study. For example, despite translations being available, no Spanish patients were recruited to the study. This likely represented the logistical challenges related to recruitment in non-English-speaking regions which we have addressed above.

Undoubtedly, achieving high-quality and accurate translations is resource intensive. The process can take time if undertaken by healthcare professionals or pose significant financial costs if study groups employ professional services. However, restricting a consensus-seeking process in the development of an international COS to a single language exposes studies to the risk of excluding important opinion. It may therefore be argued that for a COS to be truly regarded as ‘international’, the consensus-seeking process should be undertaken in the native language of the participant. Whilst we have demonstrated that non-English Delphi surveys coupled with local study promotion can increase the number of total participants, it is not known whether these additional participants bring a different perspective that has not already been captured through the English-language version. It is likely that there are many additional factors which may contribute to the validity of Delphi survey results (e.g. cultural and geographical differences of participants) and COS developers should consider these carefully during the planning phase. These, along with other factors, will be the focus of a future analysis by our study group.

### Strengths, limitations and implications for methodological practice

We have demonstrated that there is no standardised approach to translation in this field. Each of the four COS groups reviewed in this paper used different methods to forward translate and utilised translation teams with different member characteristics. A strength of our approach is that it is based on international consensus guidelines and was easily reproduced in several culturally diverse regions. We also provided detailed justifications for each step we adopted and the pragmatic adaptations which will help other COS developers, particularly those who may be limited with respect to financial resources. Our recommendation is therefore that COS developers should consider adopting this approach alongside other important considerations to broaden recruitment to their Delphi surveys.

Limitations of our study include that it focussed on examining the current translation methodology used in Delphi surveys for COS. It is possible that a broader review of survey translations could have yielded a greater understanding of current approaches to translations and methodological aspects that have not been accounted for. Furthermore, it can be argued that the process by which the ISPOR-TCA consensus guidelines were adapted to meet our needs was not undertaken using a formalised approach [20]. We opted to use an informal approach to guideline adaptation due to the limited resources available to us. Furthermore, whilst we have translated our survey into 7 target languages, this may not have been a sufficient number needed for a COS. However, it may be argued that the need for such broad participation of stakeholders in a Delphi survey is not necessary and that more targeted recruitment of individuals is sufficient, negating the requirement for the approaches discussed. This is an area which will be examined as part of our future work.

## Conclusion

We present a method of translating Delphi surveys for use in the development of COS adapted from international consensus guidelines in the field of outcome reporting. Consideration of the issues described will improve planning by other COS developers and can be used to widen international participation from both patients and healthcare professionals. Ultimately, internationally developed COS will improve the relevance of the core set to large-scale clinical trials and therefore improve healthcare decision-making.

## Supplementary Information

**Additional file 1.** Translation Methodology Questionnaire.

**Additional file 2.** Instructions for translating files related to the GASTROS Delphi Survey.

**Additional file 3.** Outcomes for translation.

**Additional file 4.** Survey presented to participants in round 1 of the Delphi.

## Data Availability

The datasets analysed during the current study are available from the corresponding author on reasonable request.

## Abbreviations

COS: Core outcome set
GASTROS: GAstric Cancer Surgery TRials Reported Outcome Standardisation
IWG: International working group
SAG: Study advisory group
ISPOR-TCA: The Professional Society for Health Economics and Outcomes Research – Translation and Cultural Adaptation group
OMERACT: Outcome Measures in Rheumatology
