# Supplementary material for: Methods for conducting international Delphi surveys to optimise global participation in core outcome set development: a case study in gastric cancer informed by a comprehensive literature review
Source: Trials. 2021 Jun 21;22:410. doi: 10.1186/s13063-021-05338-x (PMC8218463; doi:10.1186/s13063-021-05338-x)
Supplement: Supplementary file 1 — Additional file 1. Translation Methodology Questionnaire. [file 13063_2021_5338_MOESM1_ESM.docx]

## Additional file 1. Translation Methodology Questionnaire

**Translations in Delphi Surveys for Core Outcome Set Development**

Thank you for answering this short survey on using translations in Delphi surveys for core outcome set development. We are developing a core outcome set for surgical trials in gastric cancer and would like to learn from your experience.

1. Name

___________________________________________

2. Name/topic of your core outcome set study

___________________________________________

3. What was the primary language of your Delphi survey?

___________________________________________

4. Which languages did you translate your Delphi survey into?

___________________________________________

5. How many participants completed all versions of the survey?

___________________________________________

6. How many participants completed the translated version of the Delphi survey?

___________________________________________

**Translation Methodology**

7. What methodological approach did you base your translations on? Please provide any references as appropriate.

___________________________________________

8. Did you use a professional translation service?

🞐 Yes

🞐 No

9. How many 'forward' translations of the 'outcomes' presented in the Delphi survey were undertaken?

🞐 1

🞐 2

🞐 More than 2

10. Which of the following best describes the 'forward' translator(s) used for your survey? Please tick all relevant options.

🞐 Professional translator

🞐 Independent from the study team

🞐 Healthcare professional

🞐 Non healthcare professional

🞐 Target language is their first language

🞐 Previous experience of medical or patient-reported outcome translation

🞐 Reside in the country of target language

🞐 Fluent in the source language

🞐 Not sure

11. How many 'backward' translations of the 'outcomes' presented in the Delphi survey were undertaken?

p 0

🞐 1

🞐 2

🞐 More than 2

**Backward Translation**

12. Which of the following best describes the ‘backward’ translators used for your survey? Please tick all relevant options.

🞐 Independent from the study team

🞐 Professional translator

🞐 Healthcare professional

🞐 Non healthcare professional

🞐 Source language is their first language

🞐 They have not seen the source document which was ‘forward translated’

🞐 Not sure

**Discrepancies & Harmonization**

13. Please summarize how was a final version of the translation agreed?

___________________________________________

14. Please summarize what plans were in place to deal with discrepancies or disputes with translations?

___________________________________________

**Cognitive Debriefing**

15. 'Cognitive debriefing' involves piloting the survey on a small group of stakeholders to test alternative wording and to check understandability, interpretation, and cultural relevance of the translation. Was this undertaken?

🞐 Yes

🞐 No

16. Who did the cognitive debriefing exercise involve? Please tick all relevant options.

🞐 Healthcare professional(s)

🞐 Patient(s)

🞐 Member(s) of the study team

🞐 Other…
