# Supplementary material for: Methods for conducting international Delphi surveys to optimise global participation in core outcome set development: a case study in gastric cancer informed by a comprehensive literature review
Source: Trials. 2021 Jun 21;22:410. doi: 10.1186/s13063-021-05338-x (PMC8218463; doi:10.1186/s13063-021-05338-x)
Supplement: Supplementary file 2 — Additional file 2. Instructions for translating files related to the GASTROS Delphi Survey. [file 13063_2021_5338_MOESM2_ESM.docx]

## Additional file 2. Instructions for translating files related to the GASTROS Delphi Survey

**Introduction**

Thank you for agreeing to manage the translation of the GASTROS Delphi survey into your local language. The aim is to develop a translation which is clear and understandable to patients, oncology specialist nurses and surgeons.

The survey has two rounds. Following rounds 1 and 2, additional translations will be required and are detailed in section 6 of this document.

There are two types of documents which will require translation; one group requiring only one forward translation (‘1FT’ - from English to the target language) and another group requiring two independent forward translations and an additional backward translation (‘2F1BT’ - from the target back to English). This document outlines the methodology to be used for each type of translation.

**All material during each step of the translation process should be kept and submitted to the chief investigator (CI), Dr Bilal Alkhaffaf, at the end of the process.**

**All materials related to this study are confidential and may not be shared under any circumstances without written consent from the CI.**

Time-frame for translations

The time to complete all tasks associated with this translation work is *1 month* from the time you receive the source documents.

Team members required for the translation

Three types of members will be needed for the translation process, each with specific characteristics which are required to comply with methodological guidelines for translations in this field^[[1]](#footnote-1)^.

- 1. **GASTROS International working group collaborator**

The collaborator is responsible for overseeing the translation of the Delphi survey and associated supporting documents with support from the CI. The collaborator is a research-active surgeon who manages gastric cancer and adheres to the ‘terms of reference’ document (agreed to prior to their involvement in the GASTROS study). The collaborator should have the following additional characteristics:

- Native speaker of the target language
- Fluent in the English language
- Resides in the target country
  1. **Forward translator(s)**

We would advise that there is one lead forward translator. The collaborator may take on this role if they deem it appropriate. Forward translators should have the following characteristics:

- Native speaker of the target language
- Fluent in the English language
- Reside in the target country
- Familiar with medical terms used to describe outcomes, preferably with previous experience in translating outcomes (although this is not mandatory).
  1. **Backward translator**

The backward translator should have the following characteristics:

- Fluent in the English-language.
- Fluent in the target language

Translation of documents only requiring one forward translation (1FT)

The following files require 1FT:

- *GASTROS Delphi survey – PIS Version 2 290518*
- *GASTROS Delphi survey – static text Version 2 220918*

There are two steps to this process:

*Forward Translation*

**What is involved?**

The source text is translated into the target language using the same layout and formatting where appropriate.

**Who performs this step?**

A single forward translator as described in section 3.2.

Dual proofreading & final verification

**What is involved?**

The translation is proofread for accuracy and quality. The proof-readers do not perform the original translation of the source file(s). Any corrections or amendments in the translation is undertaken through discussion between the translator and proof-readers.

Any content found to be missing from the existing translation undergoes standard translation and separate proofreading steps.

**Who performs this step?**

Two separate persons fluent in English and native in the target language. This may be the international collaborator and a second forward translator as set out in sections 3.1 and 3.2.

Translation of documents requiring two forward and one backward translation (2F1BT)

*The following files require 2F1BT:*

GASTROS Delphi survey – user-defined text

GASTROS Delphi survey – outcomes

The methodology used for this translation is based on consensus guidelines as set out by the International Society for Pharmacoeconomics and Outcomes Research (ISPOR)^1^.

*Dual Forward Translation*

**What is involved?**

The source text is translated into the target language using the templates provided.

**Who performs this step?**

Two independent forward translators as set out in section 3.2.

*Forward Translation Reconciliation*

**What is involved?**

The two forward translations are reconciled into a third consensus (“best of both”) translation by the lead forward translator. Any issues that arise from this stage are discussed with the international collaborator and CI (as appropriate) and the reconciliation refined if necessary.

*Single Back Translation*

**What is involved?**

The forward translation is back translated into English.

**Who performs this step?**

A single back translator as set out in section 3.3.

Back Translation Review

**What is involved?**

The back translation is reviewed against the original source document.

**Who performs this step?**

The international collaborator: any issues arising from this review are passed to the lead forward translator for comment. Where appropriate, the lead translator provides alternative wordings (along with their own back translation) to get closer to achieving conceptual equivalence with the original English. Where necessary, support and advice from the study CI should be sought.

*Piloting and review of the online survey*

**What is involved?**

The translated texts are provided to the CI, who will organise for a pilot Delphi survey to be compiled.

**Who performs this step?**

The pilot survey is undertaken by the international collaborator. Any issues with wording, comprehension and formatting are highlighted and further discussion and refinement is made by the international collaborator in conjunction with the translation team and CI.

*Additional translations*

The translation work described above will enable participants to complete round 1 of the survey. Further translations will be required during the following stages of the study:

After completion of round 1 of the survey

**Additional Outcomes**

During round 1 of the survey, participants will be given the opportunity to add outcomes that they believe should be considered by participants in round 2. Any additional outcomes will require dual translation (target language to English) and proof-reading using the methodology as set out in section 4 of this document.

Should the GASTROS research team believe that a new outcome has been identified by a participant, this additional outcome will require a 2F1BT translation as outlined above.

**Chart Legends**

All survey responses will be analysed following round 1. These responses will be presented to participants in round 2 where they will have the opportunity to re-score all the outcomes again (plus any additional outcomes). The responses from round 1 will be presented using histogram charts which will require translation.

After completion of round 2

Participants will be given the opportunity to provide a reason why they changed their score from round 1 (if applicable). Responses will require dual translation (target language to English) and proof-reading using the methodology as set out in section 4 of this document.

Further information

If you require any further clarification or information, please do not hesitate to contact…

1. Wild D, Grove A, Martin M, Eremenco S, McElroy S, Verjee-Lorenz A, Erikson P; ISPOR Task Force for Translation and Cultural Adaptation. Principles of Good Practice for the Translation and Cultural Adaptation Process for Patient-Reported Outcomes (PRO) Measures: report of the ISPOR Task Force for Translation and Cultural Adaptation. Value Health. 2005 Mar-Apr;8(2):94-104. [↑](#footnote-ref-1)
