# Supplementary material for: Methods for conducting international Delphi surveys to optimise global participation in core outcome set development: a case study in gastric cancer informed by a comprehensive literature review
Source: Trials. 2021 Jun 21;22:410. doi: 10.1186/s13063-021-05338-x (PMC8218463; doi:10.1186/s13063-021-05338-x)
Supplement: Supplementary file 3 — Additional file 3. Outcomes for translation. [file 13063_2021_5338_MOESM3_ESM.docx]

## Additional file 3. Outcomes for translation.

| **Outcome** | **Plain Language Description** | **Domain** |
| --- | --- | --- |
| **Disease-free survival** | How long someone is alive without cancer returning. | Outcomes Related to Death |
| **Dying from stomach cancer** | Dying from stomach cancer. This does not include dying from treatment for stomach cancer. | Outcomes Related to Death |
| **Dying from any cause** | Dying from any cause. This includes dying from treatment for stomach cancer. | Outcomes Related to Death |
| **Surgery-related death** | Dying as a direct consequence of surgery | Outcomes Related to Death |
| **Cardiac complications** | Complications related to the heart, such as a heart attack or abnormal heart rhythms. | Physiological & Clinical Outcomes |
| **Endocrine complications** | Complications related to the body’s hormones, such as developing diabetes. | Physiological & Clinical Outcomes |
| **Anastomotic complications** | Complications related to surgical joins made as a result of removing stomach cancer. | Physiological & Clinical Outcomes |
| **Gastro-intestinal functional problems** | Symptoms related to how the digestive system works, including those which may become problematic months after discharge from hospital. | Physiological & Clinical Outcomes |
| **Bowel Complications** | Problems with the bowel, such as those which occur while still in hospital (not including anastomotic complications). | Physiological & Clinical Outcomes |
| **Time to recommencing oral intake** | The time taken for a patient’s bowel function to return after surgery, such that they can start eating and drinking again. | Physiological & Clinical Outcomes |
| **Fatigue** | Feeling of tiredness. | Physiological & Clinical Outcomes |
| **Multiple organ failure** | A severe complication which leads to several organs (such as the heart or lungs) not functioning properly. | Physiological & Clinical Outcomes |
| **Pain** |  | Physiological & Clinical Outcomes |
| **Surgical Stress Response** | The body’s response to the stress of surgery. | Physiological & Clinical Outcomes |
| **Gallbladder complications** | Complications related to the gallbladder. | Physiological & Clinical Outcomes |
| **Hepatic Complications** | Complications related to the liver. | Physiological & Clinical Outcomes |
| **Pancreatic Complications** | Complications related to the pancreas. | Physiological & Clinical Outcomes |
| **Abdominal Collection** | Fluid or infections in the abdomen. | Physiological & Clinical Outcomes |
| **Other infections** | General infections which are not related to the abdomen, lungs or wounds. | Physiological & Clinical Outcomes |
| **Nutritional Effects** | The extent to which the body can consume and use the nutrients needed to function properly. | Physiological & Clinical Outcomes |
| **Recurrence of Cancer** | The chances of the cancer coming back. | Physiological & Clinical Outcomes |
| **Renal complications** | Complications related to the kidneys, such as kidney failure. | Physiological & Clinical Outcomes |
| **Urinary complications** | Complications related to the bladder and urinary tract, such as a urinary infection. | Physiological & Clinical Outcomes |
| **Post-operative psychosis** | A temporary altered mental state after surgery which includes not being able to tell what is or isn’t real. | Physiological & Clinical Outcomes |
| **Respiratory complications** | Complications such as a chest infection, a collapsed lung or fluid on the lungs. | Physiological & Clinical Outcomes |
| **Wound complications** | Problems with the surgical incisions, including infection and problems with healing. | Physiological & Clinical Outcomes |
| **Cerebro-vascular complications** | Complications such as strokes and mini-strokes. | Physiological & Clinical Outcomes |
| **Thrombo-embolic complications** | Complications such as blood-clots in the legs and lungs. | Physiological & Clinical Outcomes |
| **Bleeding** | Blood loss as a result of surgery | Physiological & Clinical Outcomes |
| **Ability to undertake physical activities** | Ability to undertake day-to-day activities including exercise | Life Impact |
| **Insomnia** | Problems with sleeping. | Life Impact |
| **Impact on sexual function** | The effect of surgery on a patient’s sexual activity. | Life Impact |
| **Ability to eat socially** | Ability to eat with friends and family. | Life Impact |
| **Ability to interact socially** | The ability to have relationships with family and friends. | Life Impact |
| **Impact of surgery on social and work roles** | The effect of surgery on being able to work and caring for others. | Life Impact |
| **Impact on mental health** | The effect of surgery on a patient’s psychological well-being. | Life Impact |
| **Impact on Physical Appearance** | The effect of surgery on a patient’s physical appearance | Life Impact |
| **Impact on cognitive functioning** | The effect of surgery on concentration and memory. | Life Impact |
| **Impact on spirituality or faith** | The effect of surgery on a patient’s spirituality or faith. | Life Impact |
| **Overall quality of life** | An overall measure of how a person’s general wellbeing has been affected by surgery. | Life Impact |
| **Impact on perception of physical health** | How healthy a patient believes they are following surgery. | Life Impact |
| **Ability to complete treatment pathway.** | Being well enough to complete all aspects of treatment, such as chemotherapy and/or radiotherapy following surgery. | Life Impact |
| **Completeness of tumour removal** | Ensuring that the tumour has been surgically removed. | Life Impact |
| **Conversion to open surgery** | The surgical team having to unexpectedly change the approach from a minimally invasive (laparoscopic or key-hole) operation to a traditional open approach, usually involving a larger incision. | Life Impact |
| **Duration of surgery** | The length of time taken to perform the surgery. | Life Impact |
| **Wound size** | The size of the wound or wounds needed to perform the surgery. | Life Impact |
| **Cost** | The overall cost of surgery. | Resource Use |
| **Duration of hospital stay** | How long a patient stays in hospital. | Resource Use |
| **Readmission to hospital** | Whether a patient needs to return to hospital after being discharged following surgery. | Resource Use |
| **Destination on Discharge** | The location where a patient is discharged to from hospital. | Resource Use |
| **Need for an additional intervention.** | Unexpected additional procedures or surgeries which may be required. | Resource Use |
| **Need for pain relief** | The need for a patient to take or be given pain relief after surgery. | Resource Use |
| **Adverse drug reaction** | Complications related to medications. | Adverse Events |
| **All-cause complications** | Any complication which may arise after surgery. | Adverse Events |
| **Intra-operative complications** | Complications which occur during surgery such as accidental injury to an organ. | Adverse Events |
| **Anaesthetic complications** | Complications specifically related to anaesthesia. | Adverse Events |
