# Supplementary material for: Methods for conducting international Delphi surveys to optimise global participation in core outcome set development: a case study in gastric cancer informed by a comprehensive literature review
Source: Trials. 2021 Jun 21;22:410. doi: 10.1186/s13063-021-05338-x (PMC8218463; doi:10.1186/s13063-021-05338-x)
Supplement: Supplementary file 4 — Additional file 4. Survey presented to participants in round 1 of the Delphi. [file 13063_2021_5338_MOESM4_ESM.docx]

## Additional file 4. Survey presented to participants in round 1 of the Delphi.


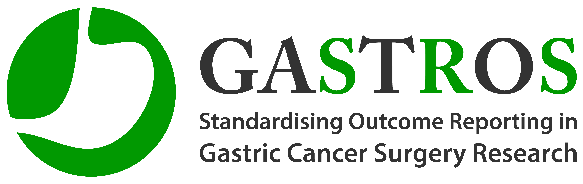


**PAPER-BASED DELPHI SURVEY FOR PATIENTS**

**MULTI-LANGUAGE VERSION 1 – 18^th^ March 2019**

**Instructions for completing the questionnaire**

You will be shown a list of outcomes. Each outcome will have a description next to it. Some outcomes also have help text with more detail.

For each outcome, you are asked to score how important it is that the outcome is included in the final core outcome set. The score is on a scale from 1 (not important) to 9 (critically important).

Your score should reflect how important **you** think it is that the outcome be measured. This is **not** an exercise to determine *how* the outcomes should be measured. The practicalities of how an outcome can be measured should **not** influence your score at this stage. How the outcomes within a core outcome set should be measured is addressed at a later stage.

The Delphi Survey includes 2 rounds. In the second round, we will ask you to review the scores you gave each outcome in the first round. You will also be able to see the group scores of surgeons, nurses and patients. You can then choose to change your score or leave it the same.

**Round 1 will be open for approximately 2-4 months. Round 2 of the survey will be open for approximately 2 months. It is extremely important that everyone who completes round 1 also completes round 2. We will contact you when it is time to complete round 2.**

**REGISTRATION DATA**

**Name or identifier: _________________________________________**

**Gender:** 🞏 Male 🞏 Female

**Age (years): _____**

**Which country do you live in? _______________________________**

**Who do you live with?**

🞏 I live alone 🞏 I live with a spouse or partner 🞏 I live with my parents 🞏 I live with my children 🞏 Other

**How long ago was your surgery (in years)? _____**

**What type of surgery did you undergo?**

🞏 Laparoscopic (keyhole) surgery 🞏 Open surgery

**How much of your stomach was removed during surgery?**

🞏 All of my stomach 🞏 Part of my stomach

**Did you have any treatments for gastric cancer other than surgery?**

🞏 No 🞏 Chemotherapy alone 🞏 Chemotherapy and radiotherapy 🞏 Radiotherapy alone

**Outcome Area 1: Death**

|  | **Outcome** | **Translation** | **Scoring** | | | | | | | | | |
| --- | --- | --- | --- | --- | --- | --- | --- | --- | --- | --- | --- | --- |
| **1.1.1** | **Disease-free survival**  How long someone is alive without any cancer returning. |  | Not that important | | | Important | | | Critically important | | |  |
|  |  |  | 1 | 2 | 3 | 4 | 5 | 6 | 7 | 8 | 9 | Unsure or  Unable to score |
| **1.1.2** | **Dying from stomach cancer**  Dying from stomach cancer. This does not include dying from treatment for stomach cancer. |  | Not that important | | | Important | | | Critically important | | |  |
|  |  |  | 1 | 2 | 3 | 4 | 5 | 6 | 7 | 8 | 9 | Unsure or  Unable to score |
| **1.1.3** | **Dying from any cause**  Dying from any cause. This includes dying from treatment for stomach cancer. |  | Not that important | | | Important | | | Critically important | | |  |
|  |  |  | 1 | 2 | 3 | 4 | 5 | 6 | 7 | 8 | 9 | Unsure or  Unable to score |
| **1.1.4** | **Surgery-related death**  Dying as a direct consequence of surgery. |  | Not that important | | | Important | | | Critically important | | |  |
|  |  |  | 1 | 2 | 3 | 4 | 5 | 6 | 7 | 8 | 9 | Unsure or  Unable to score |

**Outcome Area 2: Physiological/Clinical Outcomes**

|  | **Outcome** | **Description** | **Scoring** | | | | | | | | | |
| --- | --- | --- | --- | --- | --- | --- | --- | --- | --- | --- | --- | --- |
| **2.3.1** | **Cardiac complications**  Complications related to the heart, such as a heart attack or abnormal heart rhythms. |  | Not that important | | | Important | | | Critically important | | |  |
|  |  |  | 1 | 2 | 3 | 4 | 5 | 6 | 7 | 8 | 9 | Unsure or  Unable to score |
| **2.4.1** | **Endocrine complications**  Complications related to the body’s hormones, such as developing diabetes. |  | Not that important | | | Important | | | Critically important | | |  |
|  |  |  | 1 | 2 | 3 | 4 | 5 | 6 | 7 | 8 | 9 | Unsure or  Unable to score |
| **2.8.1** | **Anastomotic complications**  Complications related to surgical joins made as a result of removing stomach cancer. |  | Not that important | | | Important | | | Critically important | | |  |
|  |  |  | 1 | 2 | 3 | 4 | 5 | 6 | 7 | 8 | 9 | Unsure or  Unable to score |
| **2.8.2** | **Gastro-intestinal functional problems**  Symptoms related to how the digestive system works, including those which may become problematic months after discharge from hospital. |  | Not that important | | | Important | | | Critically important | | |  |
|  |  |  | 1 | 2 | 3 | 4 | 5 | 6 | 7 | 8 | 9 | Unsure or  Unable to score |
| **2.8.3** | **Bowel Complications**  Problems with the bowel, such as those which occur while still in hospital (not including anastomotic complications). |  | Not that important | | | Important | | | Critically important | | |  |
|  |  |  | 1 | 2 | 3 | 4 | 5 | 6 | 7 | 8 | 9 | Unsure or  Unable to score |

|  | **Outcome** | **Description** | **Scoring** | | | | | | | | | |
| --- | --- | --- | --- | --- | --- | --- | --- | --- | --- | --- | --- | --- |
| **2.8.4** | **Time to recommencing oral intake**  The time taken for a patient’s bowel function to return after surgery, such that they can start eating and drinking again. |  | Not that important | | | Important | | | Critically important | | |  |
|  |  |  | 1 | 2 | 3 | 4 | 5 | 6 | 7 | 8 | 9 | Unsure or  Unable to score |
| **2.9.1** | **Fatigue**  Feeling of tiredness. |  | Not that important | | | Important | | | Critically important | | |  |
|  |  |  | 1 | 2 | 3 | 4 | 5 | 6 | 7 | 8 | 9 | Unsure or  Unable to score |
| **2.9.2** | **Multiple organ failure**  A severe complication which leads to several organs (such as the heart or lungs) not functioning properly. |  | Not that important | | | Important | | | Critically important | | |  |
|  |  |  | 1 | 2 | 3 | 4 | 5 | 6 | 7 | 8 | 9 | Unsure or  Unable to score |
| **2.9.3** | **Pain**  A severe complication which leads to several organs (such as the heart or lungs) not functioning properly. |  | Not that important | | | Important | | | Critically important | | |  |
|  |  |  | 1 | 2 | 3 | 4 | 5 | 6 | 7 | 8 | 9 | Unsure or  Unable to score |
| **2.9.4** | **Surgical Stress Response**  The body’s response to the stress of surgery. |  | Not that important | | | Important | | | Critically important | | |  |
|  |  |  | 1 | 2 | 3 | 4 | 5 | 6 | 7 | 8 | 9 | Unsure or  Unable to score |
| **2.10.1** | **Gallbladder complications**  Complications related to the gallbladder. |  | Not that important | | | Important | | | Critically important | | |  |
|  |  |  | 1 | 2 | 3 | 4 | 5 | 6 | 7 | 8 | 9 | Unsure or  Unable to score |
|  | **Outcome** | **Description** | **Scoring** | | | | | | | | | |
| **2.10.2** | **Hepatic Complications**  Complications related to the liver. |  | Not that important | | | Important | | | Critically important | | |  |
|  |  |  | 1 | 2 | 3 | 4 | 5 | 6 | 7 | 8 | 9 | Unsure or  Unable to score |
| **2.10.3** | **Pancreatic Complications**  Complications related to the pancreas. |  | Not that important | | | Important | | | Critically important | | |  |
|  |  |  | 1 | 2 | 3 | 4 | 5 | 6 | 7 | 8 | 9 | Unsure or  Unable to score |
| **2.12.1** | **Abdominal Collection**  Fluid or infections in the abdomen. |  | Not that important | | | Important | | | Critically important | | |  |
|  |  |  | 1 | 2 | 3 | 4 | 5 | 6 | 7 | 8 | 9 | Unsure or  Unable to score |
| **2.12.2** | **Other infections**  General infections which are not related to the abdomen, lungs or wounds. |  | Not that important | | | Important | | | Critically important | | |  |
|  |  |  | 1 | 2 | 3 | 4 | 5 | 6 | 7 | 8 | 9 | Unsure or  Unable to score |
| **2.14.1** | **Nutritional Effects**  The extent to which the body can consume and use the nutrients needed to function properly. |  | Not that important | | | Important | | | Critically important | | |  |
|  |  |  | 1 | 2 | 3 | 4 | 5 | 6 | 7 | 8 | 9 | Unsure or  Unable to score |
| **2.16.1** | **Recurrence of Cancer**  The chances of the cancer coming back. |  | Not that important | | | Important | | | Critically important | | |  |
|  |  |  | 1 | 2 | 3 | 4 | 5 | 6 | 7 | 8 | 9 | Unsure or  Unable to score |
|  | **Outcome** | **Description** | **Scoring** | | | | | | | | | |
| **2.19.1** | **Renal complications**  Complications related to the kidneys, such as kidney failure. |  | Not that important | | | Important | | | Critically important | | |  |
|  |  |  | 1 | 2 | 3 | 4 | 5 | 6 | 7 | 8 | 9 | Unsure or  Unable to score |
| **2.19.2** | **Urinary complications**  Complications related to the bladder and urinary tract, such as a urinary infection. |  | Not that important | | | Important | | | Critically important | | |  |
|  |  |  | 1 | 2 | 3 | 4 | 5 | 6 | 7 | 8 | 9 | Unsure or  Unable to score |
| **2.21.1** | **Post-operative psychosis**  A temporary altered mental state after surgery which includes not being able to tell what is or isn’t real. |  | Not that important | | | Important | | | Critically important | | |  |
|  |  |  | 1 | 2 | 3 | 4 | 5 | 6 | 7 | 8 | 9 | Unsure or  Unable to score |
| **2.22.1** | **Respiratory complications**  Complications such as a chest infection, a collapsed lung or fluid on the lungs. |  | Not that important | | | Important | | | Critically important | | |  |
|  |  |  | 1 | 2 | 3 | 4 | 5 | 6 | 7 | 8 | 9 | Unsure or  Unable to score |
| **2.23.1** | **Wound complications**  Problems with the surgical incisions, including infection and problems with healing. |  | Not that important | | | Important | | | Critically important | | |  |
|  |  |  | 1 | 2 | 3 | 4 | 5 | 6 | 7 | 8 | 9 | Unsure or  Unable to score |
| **2.24.1** | **Cerebro-vascular complications**  Complications such as strokes and mini-strokes. |  | Not that important | | | Important | | | Critically important | | |  |
|  |  |  | 1 | 2 | 3 | 4 | 5 | 6 | 7 | 8 | 9 | Unsure or  Unable to score |

|  | **Outcome** | **Description** | **Scoring** | | | | | | | | | |
| --- | --- | --- | --- | --- | --- | --- | --- | --- | --- | --- | --- | --- |
| **2.24.2** | **Thrombo-embolic complications**  Complications such as blood-clots in the legs and lungs. |  | Not that important | | | Important | | | Critically important | | |  |
|  |  |  | 1 | 2 | 3 | 4 | 5 | 6 | 7 | 8 | 9 | Unsure or  Unable to score |
| **2.24.3** | **Bleeding**  Blood loss as a result of surgery. |  | Not that important | | | Important | | | Critically important | | |  |
|  |  |  | 1 | 2 | 3 | 4 | 5 | 6 | 7 | 8 | 9 | Unsure or  Unable to score |

**Outcome Area 3: Life Impact**

|  | **Outcome** | **Description** | **Scoring** | | | | | | | | | |
| --- | --- | --- | --- | --- | --- | --- | --- | --- | --- | --- | --- | --- |
| **3.25.1** | **Ability to undertake physical activities**  Ability to undertake day-to-day activities including exercise. |  | Not that important | | | Important | | | Critically important | | |  |
|  |  |  | 1 | 2 | 3 | 4 | 5 | 6 | 7 | 8 | 9 | Unsure or  Unable to score |
| **3.25.2** | **Insomnia**  Problems with sleeping. |  | Not that important | | | Important | | | Critically important | | |  |
|  |  |  | 1 | 2 | 3 | 4 | 5 | 6 | 7 | 8 | 9 | Unsure or  Unable to score |

|  | **Outcome** | **Description** | **Scoring** | | | | | | | | | |
| --- | --- | --- | --- | --- | --- | --- | --- | --- | --- | --- | --- | --- |
| **3.25.3** | **Impact on sexual function**  The effect of surgery on a patient’s sexual activity. |  | Not that important | | | Important | | | Critically important | | |  |
|  |  |  | 1 | 2 | 3 | 4 | 5 | 6 | 7 | 8 | 9 | Unsure or  Unable to score |
| **3.26.1** | **Ability to eat socially**  Ability to eat with friends and family. |  | Not that important | | | Important | | | Critically important | | |  |
|  |  |  | 1 | 2 | 3 | 4 | 5 | 6 | 7 | 8 | 9 | Unsure or  Unable to score |
| **3.26.2** | **Ability to interact socially**  The ability to have relationships with family and friends. |  | Not that important | | | Important | | | Critically important | | |  |
|  |  |  | 1 | 2 | 3 | 4 | 5 | 6 | 7 | 8 | 9 | Unsure or  Unable to score |
| **3.27.1** | **Impact of surgery on social and work roles**  The effect of surgery on being able to work and caring for others. |  | Not that important | | | Important | | | Critically important | | |  |
|  |  |  | 1 | 2 | 3 | 4 | 5 | 6 | 7 | 8 | 9 | Unsure or  Unable to score |
| **3.28.1** | **Impact on mental health**  The effect of surgery on a patient’s psychological well-being. |  | Not that important | | | Important | | | Critically important | | |  |
|  |  |  | 1 | 2 | 3 | 4 | 5 | 6 | 7 | 8 | 9 | Unsure or  Unable to score |
| **3.28.2** | **Impact on Physical Appearance**  The effect of surgery on a patient’s physical appearance |  | Not that important | | | Important | | | Critically important | | |  |
|  |  |  | 1 | 2 | 3 | 4 | 5 | 6 | 7 | 8 | 9 | Unsure or  Unable to score |
|  | **Outcome** | **Description** | **Scoring** | | | | | | | | | |
| **3.29.1** | **Impact on cognitive functioning**  The effect of surgery on concentration and memory. |  | Not that important | | | Important | | | Critically important | | |  |
|  |  |  | 1 | 2 | 3 | 4 | 5 | 6 | 7 | 8 | 9 | Unsure or  Unable to score |
| **3.29.2** | **Impact on spirituality or faith**  The effect of surgery on a patient’s spirituality or faith. |  | Not that important | | | Important | | | Critically important | | |  |
|  |  |  | 1 | 2 | 3 | 4 | 5 | 6 | 7 | 8 | 9 | Unsure or  Unable to score |
| **3.30.1** | **Overall quality of life**  An overall measure of how a person’s general wellbeing has been affected by surgery. |  | Not that important | | | Important | | | Critically important | | |  |
|  |  |  | 1 | 2 | 3 | 4 | 5 | 6 | 7 | 8 | 9 | Unsure or  Unable to score |
| **3.31.1** | **Impact on perception of physical health**  How healthy a patient believes they are following surgery. |  | Not that important | | | Important | | | Critically important | | |  |
|  |  |  | 1 | 2 | 3 | 4 | 5 | 6 | 7 | 8 | 9 | Unsure or  Unable to score |
| **3.32.1** | **Ability to complete treatment pathway.**  Being well enough to complete all aspects of treatment, such as chemotherapy and/or radiotherapy following surgery. |  | Not that important | | | Important | | | Critically important | | |  |
|  |  |  | 1 | 2 | 3 | 4 | 5 | 6 | 7 | 8 | 9 | Unsure or  Unable to score |
| **3.32.2** | **Completeness of tumour removal**  Ensuring that the tumour has been surgically removed. |  | Not that important | | | Important | | | Critically important | | |  |
|  |  |  | 1 | 2 | 3 | 4 | 5 | 6 | 7 | 8 | 9 | Unsure or  Unable to score |

|  | **Outcome** | **Description** | **Scoring** | | | | | | | | | |
| --- | --- | --- | --- | --- | --- | --- | --- | --- | --- | --- | --- | --- |
| **3.32.3** | **Conversion to open surgery**  The surgical team having to unexpectedly change the approach from a minimally invasive (laparoscopic or key-hole) operation to a traditional open approach, usually involving a larger incision. |  | Not that important | | | Important | | | Critically important | | |  |
|  |  |  | 1 | 2 | 3 | 4 | 5 | 6 | 7 | 8 | 9 | Unsure or  Unable to score |
| **3.32.4** | **Duration of surgery**  The length of time taken to perform the surgery. |  | Not that important | | | Important | | | Critically important | | |  |
|  |  |  | 1 | 2 | 3 | 4 | 5 | 6 | 7 | 8 | 9 | Unsure or  Unable to score |
| **3.32.5** | **Wound size**  The size of the wound or wounds needed to perform the surgery. |  | Not that important | | | Important | | | Critically important | | |  |
|  |  |  | 1 | 2 | 3 | 4 | 5 | 6 | 7 | 8 | 9 | Unsure or  Unable to score |

**Outcome Area 4: Resource Use**

|  | **Outcome** | **Description** | **Scoring** | | | | | | | | | |
| --- | --- | --- | --- | --- | --- | --- | --- | --- | --- | --- | --- | --- |
| **4.34.1** | **Cost**  The overall cost of surgery. |  | Not that important | | | Important | | | Critically important | | |  |
|  |  |  | 1 | 2 | 3 | 4 | 5 | 6 | 7 | 8 | 9 | Unsure or  Unable to score |
| **4.35.1** | **Duration of hospital stay**  How long a patient stays in hospital. |  | Not that important | | | Important | | | Critically important | | |  |
|  |  |  | 1 | 2 | 3 | 4 | 5 | 6 | 7 | 8 | 9 | Unsure or  Unable to score |
| **4.35.2** | **Readmission to hospital**  Whether a patient needs to return to hospital after being discharged following surgery. |  | Not that important | | | Important | | | Critically important | | |  |
|  |  |  | 1 | 2 | 3 | 4 | 5 | 6 | 7 | 8 | 9 | Unsure or  Unable to score |
| **4.35.3** | **Destination on Discharge**  The location where a patient is discharged to from hospital. |  | Not that important | | | Important | | | Critically important | | |  |
|  |  |  | 1 | 2 | 3 | 4 | 5 | 6 | 7 | 8 | 9 | Unsure or  Unable to score |
| **4.36.1** | **Need for an additional intervention.**  Unexpected additional procedures or surgeries which may be required. |  | Not that important | | | Important | | | Critically important | | |  |
|  |  |  | 1 | 2 | 3 | 4 | 5 | 6 | 7 | 8 | 9 | Unsure or  Unable to score |

|  | **Outcome** | **Description** | **Scoring** | | | | | | | | | |
| --- | --- | --- | --- | --- | --- | --- | --- | --- | --- | --- | --- | --- |
| **4.36.2** | **Need for pain relief**  The need for a patient to take or be given pain relief after surgery. |  | Not that important | | | Important | | | Critically important | | |  |
|  |  |  | 1 | 2 | 3 | 4 | 5 | 6 | 7 | 8 | 9 | Unsure or  Unable to score |

**Outcome Area 5: Adverse Events**

|  | **Outcome** | **Description** | **Scoring** | | | | | | | | | |
| --- | --- | --- | --- | --- | --- | --- | --- | --- | --- | --- | --- | --- |
| **5.38.1** | **Adverse drug reaction**  Complications related to medications. |  | Not that important | | | Important | | | Critically important | | |  |
|  |  |  | 1 | 2 | 3 | 4 | 5 | 6 | 7 | 8 | 9 | Unsure or  Unable to score |
| **5.38.2** | **All-cause complications**  Any complication which may arise after surgery. |  | Not that important | | | Important | | | Critically important | | |  |
|  |  |  | 1 | 2 | 3 | 4 | 5 | 6 | 7 | 8 | 9 | Unsure or  Unable to score |
| **5.38.3** | **Intra-operative complications**  Complications which occur during surgery such as accidental injury to an organ. |  | Not that important | | | Important | | | Critically important | | |  |
|  |  |  | 1 | 2 | 3 | 4 | 5 | 6 | 7 | 8 | 9 | Unsure or  Unable to score |
| **5.38.4** | **Anaesthetic complications**  Complications specifically related to anaesthesia. |  | Not that important | | | Important | | | Critically important | | |  |
|  |  |  | 1 | 2 | 3 | 4 | 5 | 6 | 7 | 8 | 9 | Unsure or  Unable to score |

**Additional Outcomes:**

**Comments:**
